# New functional insights from the monkey claustrum

**DOI:** 10.1093/nsr/nwag414

**Published:** 2026-07-06

**Authors:** Andreas B Wulff, Brian N Mathur

**Affiliations:** Department of Pharmacology and Physiology, School of Medicine, University of Maryland, Baltimore, MD 21201, USA; Department of Pharmacology and Physiology, School of Medicine, University of Maryland, Baltimore, MD 21201, USA; Department of Psychiatry, School of Medicine, University of Maryland, Baltimore, MD 21201, USA; University of Maryland Medicine Institute for Neuroscience Discovery, University of Maryland, Baltimore, MD 21201, USA; Kahlert Institute for Addiction Medicine, University of Maryland, Baltimore, MD 21201, USA

**Keywords:** cognitive control, cortical networks, cognitive networks, non-human primate, macaque, tract tracing, connectome, transcriptome

## Abstract

The claustrum is a subcortical nucleus with widespread, reciprocal connections to the cortex and beyond, inspiring many models of its function. Decades of work from rodents provide a foundational understanding of the anatomy and functional organization of the claustrum, which is more recently aided by human functional neuroimaging studies. However, important questions remain with broad functional implications. These center on claustrum anatomical boundaries, functional organization, and the putative expansion of human claustrum connectivity over that predicted by rodent studies alone. Providing new insights by spanning the large rodent-to-human void, a landmark study by Lei and colleagues recently provided thorough molecular and connectivity profiling of the macaque claustrum. Surprising findings of lateralized and expanded claustrum connectivity with cortical and subcortical partners help to explain human functional connectivity data and the discovery of a well-developed organization of the monkey claustrum into overlapping functional sub-domains provides deep insight into claustrum function when considering rodent synaptic connectivity data. Contextualizing these findings, we provide an elaborated model of the function of the claustrum in cognition.

## INTRODUCTION

The claustrum is a telencephalic subcortical nucleus that closely borders the striatum medially, the insular cortex laterally, and the endopiriform nucleus ventrally. Despite these appositions, the claustrum is now recognized as a distinct nucleus with unique gene expression profiles. It is composed largely of spiny, glutamatergic projection neurons expressing either vesicular glutamate transporter (vGLUT) 1 or 2 [1–3], as well as GABAergic interneurons, including parvalbumin-, somotostatin-, and vasoactive intestinal peptide-expressing subtypes [4]. While overlap exists in gene expression with cortical pyramidal neurons, claustrum projection neurons express Ntng2, Nr4a2 (Nurr1), Lxn, Oprk1, Smim32, Nr2f2, and Htr2c (see Table 1 for a complete list of gene names and associated structures); genes that are also expressed in the endopiriform nucleus, but not surrounding cortical areas [2,5–13]. The claustrum also expresses genes that are poorly expressed in neighboring structures, including Gnb4, Gng2, Cux2, Slc17a6 (vGLUT2), Egr2, and Htr2a [1,2,5,7,8,14–18]. Thus, rodent molecular expression profiles demarcate the claustrum as an independent region embedded below the deep layers of the insular cortex [14,15,19,20].

**Table 1.** A list of genetic markers specific to the claustrum, dorsal endopiriform nucleus, the region bordering the claustrum, or both the claustrum and endopiriform nucleus.

Gene symbol	Gene name	Related structure(s)
Gng2	G-protein subunit gamma 2	Claustrum
Gnb4	Guanine nucleotide-binding protein subunit beta-4	Claustrum
Cux2	Cut-like homeobox 2	Claustrum
Slc17a6 (Vglut2)	Vesicular glutamate transporter 2	Claustrum
Egr2	Early growth response 2	Claustrum
Htr2a	Serotonin receptor 2A	Claustrum
RGS6	Regulator of G-protein signaling 6	Claustrum
ZFPM2	Zinc-finger protein multitype 2	Claustrum
Smyd1	SET and MYND domain-containing 1	Claustrum
B3gat2	Beta-1,3-glucuronyltransferase 2	Claustrum
Fam198b	Family with sequence similarity 198 member B	Claustrum
Olfml2b	Olfactomedin-like 2B	Claustrum
Sostdc1	Sclerostin domain-containing 1	Claustrum
Stk32b	Serine/threonine kinase 32B	Claustrum
Fshr	Follicle stimulating hormone receptor	Claustrum
Gal	Galanin and GMAP prepropeptide	Claustrum
Tfap2d	Transcription factor AP-2 delta	Claustrum
Oas1	2'-5'-oligoadenylate synthetase 1	Claustrum
Ttn	Titin	Dorsal endopiriform nucleus
Slc17a8 (Vglut3)	Vesicular glutamate transporter 3	Dorsal endopiriform nucleus
Iqcf3	Iq motif-containing F3	Region bordering claustrum
Eaf2	Ell-associated factor 2	Region bordering claustrum
Filip1l	Filamin A-interating protein 1-like	Region bordering claustrum
Rxfp1	Relaxin family peptide receptor 1	Region bordering claustrum
Cplx3	Complexin 3	Region bordering claustrum
Cabp7	Calcium binding protein 7	Region bordering claustrum
Ctgf	Connective tissue growth factor	Region bordering claustrum
Sulf1	Sulfatase 1	Region bordering claustrum
Ntng2	Netrin-G2	Claustrum/dorsal endopiriform nucleus
Nr4a2 (Nurr1)	Nuclear receptor subfamily 4 group A member 2	Claustrum/dorsal endopiriform nucleus
Lxn	Latexin	Claustrum/dorsal endopiriform nucleus
Oprk1	Opioid receptor kappa 1	Claustrum/dorsal endopiriform nucleus
Smim32	Small integral membrane protein 32	Claustrum/dorsal endopiriform nucleus
Nr2f2 (COUP-TFII)	Nuclear receptor subfamily 2 group F member 2	Claustrum/dorsal endopiriform nucleus
Htr2C	Serotonin receptor 2C	Claustrum/dorsal endopiriform nucleus

Claustrum identity is further characterized by unique projection neuron physiological characteristics [3,4,20–23] as well as afferent and efferent connectivity profiles that are distinct from the neighboring insular cortex and endopiriform nucleus [15,24,25]. Adding to this complexity, the input-to-output map of mouse claustrum projection neurons depends on neuronal subtype [22,23]. Generally, the rodent claustrum is reciprocally connected to many areas of cortex, including prefrontal cortex, cingulate cortex, posterior parietal cortex and more weakly with sensory and motor cortices [15,26,27]. Notably, the rodent claustrum displays limited connectivity to the anterior insula [28], which itself exhibits greater subcortical connectivity compared to the claustrum [14,24]. Functionally, the rodent claustrum receives the strongest inputs from frontal cortices like the prelimbic, infralimbic and anterior cingulate cortices [23,29]. The claustrum substantially projects back to these same regions as well as other, more distant, cortical areas such as the visual cortex and the posterior parietal cortex [23,29,30].

Expansive analysis of the strength of cortico-claustro-cortical circuitries suggests that the rodent claustrum functionally mediates activity between cortical regions that act together to promote a common function. Accordingly, the rodent claustrum exhibits a functional domain organization [23,29,30]. Taken together with rodent and primate neuroimaging functional and structural connectivity data [31–37], the claustrum is widely connected with many areas of cortex and may be uniquely positioned to allow for inter-areal cortical communication via synchronous neuronal activity [38,39]. This unique anatomical architecture inspired many models of claustrum function, including multimodal sensory integration and consciousness [38], selective attention [40], network instantiation supporting cognitive control [41], and sleep [39,42,43]. Functional data from rodent and human studies have refined these models (reviewed in [41]) and consensus may be emerging, at least in waking states, around the idea that the claustrum broadly supports cognitive control [16,18,31,44–48].

With claustrum knowledge centered in rodents and humans, non-human primate studies—while present—are relatively few. To date, these studies establish that the monkey claustrum is also bidirectionally connected with several areas of cortex [49–73]. However, important, basic questions for all primates persist. First, the anatomical demarcation of the primate claustrum boundaries are historically murky. Given that unclear borders muddy connectivity data interpretation, this is a major impediment to assessing functional implications in primates. Second, human neuroimaging studies, compared to rodent tract tracing data, suggest the human claustrum more expansively connects with both cortical and subcortical sites [31,33,34]. This, together with observations of ipsi-lateralized claustrum-cortex connectivity in humans not seen as strongly in rodents, suggests evolutionary changes that require substantiation. Finally, whether the primate claustrum is functionally organized into modules with distinct input-to-output connectivity akin to rodents has remained elusive. Addressing these questions and providing deep insight into claustrum structure and function across the rodent-to-human divide is a thorough macaque study by Lei and colleagues [74].

## MACAQUE CLAUSTRUM ANATOMICAL INSIGHTS

**Figure 1.**
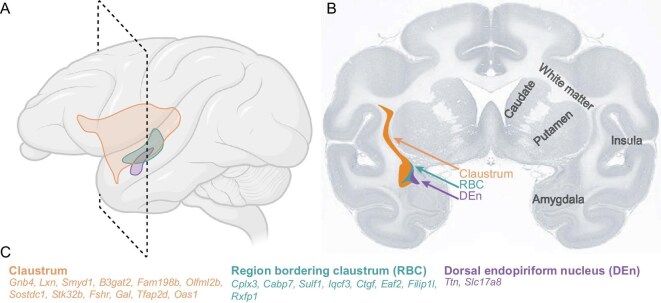
Macaque claustrum is separated from the endopiriform nucleus by the ‘region bordering claustrum’ (RBC). (A) Lateral view of the macaque brain with the territory of the claustrum (orange), region bordering claustrum (RBC, teal), and the dorsal endopiriform nucleus (DEn, purple). (B) Coronal section of the macaque brain with Nissl staining (left hemisphere is mirrored on the right). The macaque claustrum is located within the white matter between the putamen and the insular cortex and dorsal to the amygdala. Spatial transcriptomics revealed three spatially segregated aspects of the claustrum complex: The claustrum (orange) separated from the DEn (purple) by the RBC (teal). (C) Several gene markers were found to be preferentially expressed in each of these three regions (a complete list of genetic markers can be found in Table 1). Panel B is adapted from the NIH Blueprint Non-Human Primate (NHP) Atlas, NIH Contracts HHSN-271-2008-00047-C to the Allen Institute for Brain Science (Seattle, WA). Available from: http://www.blueprintnhpatlas.org/. The author’s opinions or views are not necessarily those of the NIH [147].

In primates, the claustrum is historically demarcated mediolaterally by borders with the external and extreme capsules. Ventrally, the borders with the endopiriform nucleus and amygdala are less well-defined. Addressing these issues using spatial transcriptomics, Lei and colleagues [74] found that while claustrum glutamatergic neurons share many gene transcripts with layer 5/6 insula neurons, they also selectively express Gnb4 and Lxn, which the authors used to substantiate that the claustrum is indeed a distinct structure mediolaterally bounded by the white matter of the external and extreme capsules consistent with previous findings in macaque using *in-situ* hybridization with Lxn [7]. Ventrally, they discovered the molecularly distinct ‘region bordering claustrum’ (RBC) is characterized by the expression of the Cplx3, Cabp7, and Ctgf genes. These transcripts, along with Sulf1, had previously been defined as insular layer 6b or claustrum ‘shell’ markers [11,15,20,75–77]. The RBC separates the macaque claustrum from the endopiriform nucleus (Fig. 1), the existence of which as a separate structure has been debated extensively [78]. In contrast, inhibitory interneurons within the claustrum exhibited no distinct molecular expression or distribution from that of neighboring regions. Considering these high-resolution spatial transcriptomic findings, the macaque claustrum borders are now better defined and provide clues to claustrum anatomical demarcations in the human brain. Using the spatial transcriptomic-defined borders of the macaque claustrum, Lei and co-authors [74] then assessed intra-claustral molecular and input-output circuit connectivity organization across 91 monkeys.

## CORTICAL CONNECTIVITY PROFILE OF THE MACAQUE CLAUSTRUM

Extending findings from other non-human primate studies, Lei *et al.* [74] found, using classical neuronal tract tracer injections within the claustrum and several cortical areas, that the macaque claustrum is reciprocally and broadly connected to the cortex. They found that macaque claustrum neurons project heavily to the prefrontal cortex, frontal motor cortex, cingulate cortex, parietal association cortex, occipital cortex, temporal cortex, and parietal sensory cortex. Surprisingly, Lei and colleagues found that the macaque claustrum sparsely projects to the hippocampus, a finding not observed in rodents [16,39,79] (although projections have been observed to the retrohippocampal region [15,80] as is also observed in the macaque). The macaque claustrum also receives inputs from almost all of these cortices with the strongest inputs arising from the prefrontal cortex, particularly the ventrolateral prefrontal cortex (area 12), the rostral prefrontal cortex (area 8b), the dorsolateral prefrontal cortex (area 9), and the orbitofrontal proisocortex. Outside of the strong projections from the prefrontal cortex, the claustrum receives input from the frontal motor cortex (F1 and F4), the anterior cingulate cortex (area 24c), parietal sensory cortex (Area 3), and the temporal cortex: particularly the insula, area TEav, and perirhinal cortex. The claustrum receives weaker inputs from the parietal association and occipital cortices. Surprisingly, the claustrum receives no inputs from the primary visual cortex (V1), which has otherwise been observed in many species including other primates [26,81–85].

Lei and colleagues [74] report that the macaque claustrum predominantly projects ipsilaterally and receives cortical input from ipsilateral cortex. Quantitatively, all cortical regions received >90% of inputs from the ipsilateral claustrum and outputs from most cortical regions were >90% to the ipsilateral claustrum. Exceptions to this are the frontal motor cortices (F3, F6, F7, and the proisocortical motor cortex) and the anterior cingulate cortex (area 24a–d), which still showed 75%–80% of their outputs to the ipsilateral claustrum. Inputs to the rodent claustrum specifically from the motor cortex and anterior cingulate exhibit a near complete contralateral connectivity bias [15,29,30,79,86], suggesting a shift toward lateralization during primate evolution. This increased lateralization of claustrum connectivity may help to explain the repeated observation of lateralized human claustrum activation and functional connectivity with ipsilateral cortical partners [31,33,46], and may reflect the lateralized cortical functions in primates such as language and slow-wave sleep states [39,42,43,87,88].

In rodents, limited inputs from subcortical regions to the claustrum exist comprising 10%–20% of total inputs [80]. These include the thalamic intralaminar regions, thalamic nucleus reuniens, basolateral amygdala, basal forebrain, supramammillary nucleus, dorsal raphe nucleus, and arguably the patch compartments of the striatum [79,80,89–95]. This complements the sparse claustro-subcortical projections observed in rodents [4,14,19,39]. The macaque claustrum, in contrast, exhibits more extensive reciprocal connectivity with subcortical regions. Following retrograde tracer injections into the area of the thalamus and amygdala, extensive labeling was observed in the claustrum. Sparser claustrum labeling was observed following retrograde tracer injections into the area of the caudate and putamen [74]. Similar to the rodent claustrum, the macaque claustrum receives inputs from the pulvinar nucleus of the thalamus, the thalamic ventral posterior nucleus, the thalamic intralaminar nuclei, the basolateral amygdala, basal forebrain, and dorsal raphe nucleus. Of these inputs, the strongest arises from the basolateral amygdala, containing more than half of all retrogradely labeled neurons in the subcortex. The functions of these projections are poorly explored but may reflect an evolutionary development to support subcortical and cortical communication for cognitive and behavioral engagement informed by emotional status [96].

## FUNCTIONAL DOMAIN ORGANIZATION OF THE CLAUSTRUM

**Figure 2.**
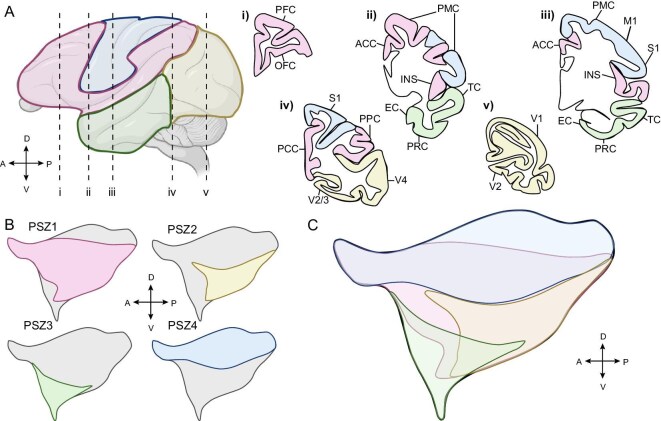
Macaque claustrum is organized into distinct cortical projection-specific zones (PSZs). (A) Cortical targets of claustrum projections can be grouped together into four cortical projection areas based on correlated spatial distribution within the claustrum. These include a target area covering the prefrontal cortex, the posterior parietal cortex, the cingulate areas, and the insula (pink), a target area covering the visual cortices (yellow), a target area covering the temporal, perirhinal and entorhinal cortices (green), and a target area covering the motor and somatosensory cortices (blue). (B) The spatial distribution of the related cortically-projecting claustrum neurons: PSZ1 neuron distribution projecting to the pink cortical zones, PSZ2 to the yellow cortical zones, PSZ3 to the green cortical zones, and PSZ4 to the blue cortical zones. (C) While PSZ2–4 occupy mutually exclusive territories of the claustrum, PSZ1 has substantial overlap with PSZ2–4. ACC, anterior cingulate cortex; EC, entorhinal cortex; INS, insula; M1, primary motor cortex; OFC, orbitofrontal cortex; PCC, posterior cingulate cortex; PFC, prefrontal cortex; PMC, premotor cortex; PPC, posterior parietal cortex; PRC, Perirhinal cortex; S1, primary sensory cortex; TC, temporal cortex; V1–4, visual cortical area 1–4.

Powerfully, Lei and colleagues [74] combined anatomical and spatial transcriptomic data to reveal ‘functional’ domains of the claustrum. Comparing spatial distribution of cortically-projecting claustrum neurons with the spatial distribution of cortical afferents into the claustrum reveals four clusters of correlated spatial distributions, which the authors named projection specific zones (PSZ)1–4 (Fig. 2). PSZ1 is connected to the anterior and posterior cingulate cortices, the dorsolateral, ventrolateral, dorsomedial, and ventromedial prefrontal cortices, and the superior and inferior parietal lobules as well as the insula and multisensory integration regions in the temporal cortex. PSZ2 is connected to the visual cortices (V1–V4) and posterior aspects of the temporal cortex. PSZ3 is connected to the entorhinal cortex, the temporal pole, and the orbitofrontal proisocortex. Finally, the PSZ4 is connected to the somatosensory cortex (Area 1–3), parietal association cortex (e.g. area 5, MIP, LIP) and frontal motor cortex (F1–5). Speculatively, the cortical regions partnered with a PSZ appear to be functionally related based on other functional studies. For instance, PSZ1 cortical regions participate in executive function (e.g. the dorsolateral prefrontal cortex and the inferior parietal lobule [97]), introspection (e.g. the medial prefrontal cortex and the posterior cingulate cortex [98]) and salience detection (e.g. the anterior cingulate cortex and the insula [99]). PSZ2 cortical regions process visual information [100]. PSZ3 cortical regions are involved in episodic memory [101,102]. PSZ4 cortical regions are involved in sensorimotor integration and motor planning [103]. While, Lei and colleagues did not perform any functional studies of the claustrum or its recipient cortical regions, the PSZs demonstrate an anatomical foundation for claustro-cortical circuits regulating wide-ranging functions.

A proto-domain organization similar to the monkey PSZ organization is observed in rodent claustrum. Neurons projecting to motor and sensory cortices are primarily located in the dorsal extent of the claustrum (sometimes referred to as the dorsal claustrum), while neurons projecting to cortices serving higher-order cognitive functions such as the retrosplenial, medial prefrontal, anterior cingulate, and parietal association cortices expand broadly and more ventrally [27,79,104]. Similarly, rodent cortical inputs to the claustrum are organized along the dorsoventral axis by sensory modality of cortical input while prefrontal cortical inputs project to the entirety of the claustrum [26]. Additionally, neurons projecting to the infralimbic and retrosplenial cortices exhibit greater excitation by infralimibic inputs compared to prelimbic prefrontal cortical inputs. Conversely, activation of prelimbic prefrontal cortical inputs induce greater excitation of neurons projecting back to the prelimbic or the parietal association cortex [23,30]. Thus, the mouse claustrum can be considered anatomically and synaptically organized into broad functional domains. The discovery of distinct PSZs in macaque suggests that functional domain organization in the claustrum is conserved across species and elaborated in primate to subserve complex inter-areal cortical control.

Beyond reciprocal connectivity with the cortex, the PSZs are also defined by differential gene expression patterns. Lei and colleagues [74] observed that certain molecular subtypes of glutamatergic claustrum neurons, as defined by their UMAP clustering, are preferentially present within distinct PSZs. For example, a subtype of the RGS6-expressing cluster of cells concentrates within PSZ1 and PSZ2, a subtype of the RGS6-expressing, two subtypes of the Gnb4-expressing, and two subtypes of the ZFPM2-expressing cluster of cells concentrate within PSZ3, and two subtypes of the Gnb4-expressing cluster of cells concentrate within PSZ4. In contrast, GABAergic neurons were equally distributed throughout the claustrum. This mirrors observations in the rodent claustrum where neurons classified based on dendritic morphology [80] or genetic expression profile [3,17,20,105] were found to have similar cortical projection targets. Taken together, this suggests that subdivisions of neurons within both the rodent and primate claustrum may share genetic, morphological and spatial characteristics to serve distinct cortical domains.

Lei and colleagues further observed that, spatially, the territory of each PSZ appears to be mutually exclusive [74]. That is, the territory of one PSZ within the claustrum does not tend to overlap with the territory of the other PSZs. Thus, claustrum neurons projecting to one functionally related set of cortical areas are not intermingled with claustrum neurons projecting to other cortical areas. Additionally, intra-claustral connectivity is bounded within these territories, thus limiting potential communication between PSZs. In mouse claustrum, intra-claustral connectivity appears to be primarily through inhibitory interneurons that are thought to filter cortical input [23,106,107], although one study found cross-modular intra-claustral connectivity in contrast to the findings in macaque claustrum [21]. Altogether, this suggests that macaque PSZs act as largely independent functional domains that may synchronize activity of distinct cortical regions. A major exception to this isolationism is the PSZ1, which spatially overlaps with the other three PSZs. This is similar to the organization of the rodent claustrum where connectivity with the prefrontal cortex is more widely distributed across the claustrum [26,27]. Given the intra-claustral connectivity data showing communication within the spatial extent of each zone, this suggests that neurons within PSZ1 intermingle and communicate with neurons in other PSZs.

## AN EXPANDED MODEL OF CLAUSTRUM FOR NETWORK INSTANTIATION IN COGNITIVE CONTROL

The extensively organized connectivity of the claustrum provides further clues as to how the claustrum supports cognition. Optimal perception and behavioral performance requires neurons in distant cortical regions to fire together [108–113]. Such synchronized co-activation of cortical regions with related computational functions are known as networks. Cortical networks carry out specialized functions such as visual processing in the visual network (consisting of V1–V4), motor planning in the sensorimotor network (consisting of the motor cortex and somatosensory cortex), and memory consolidation in the anterior-temporal network (consisting of the entorhinal cortex and the temporal pole region) [114–116]. Other cortical networks govern cognitive control functions that can be classified into three network subtypes. The first type includes the executive control network, governing interactions with environmental (i.e. extrinsic) cues for executive attention and working memory, that consists of the dorsolateral prefrontal cortex and the posterior parietal cortex [117,118]. Anti-correlated with the executive control network is the default mode network, which consists of the medial prefrontal and the posterior cingulate cortices and supports introspective (i.e. intrinsic) control [117,119–121]. Finally, the salience network, consisting of the anterior cingulate and the anterior insular cortices, detects salient stimuli to subsequently guide the activation of executive and default mode networks [118,122,123]. In comparing these cortical networks with the observed PSZ cortical projection regions, it is striking how closely the different PSZ projections align with separate cortical networks. For example, PSZ2 projects to the visual network nodes, PSZ3 projects to the anterior-temporal network nodes, and PSZ4 projects to somatomotor network nodes. Finally, PSZ1 projects to broader extrinsic and intrinsic cognitive control network nodes [74].

Cortico-claustro-cortical circuits are hypothesized to support cortical network activity. Supporting this, cortical inputs are strongest to claustrum neurons projecting to regions within the same cortical network [23]. Thus, cortical inputs are likely to activate claustrum neurons projecting to within-network cortical regions that, in turn, synchronize cortical activity in the delta and gamma frequency range [39,43,124,125]. Inputs from the anterior cingulate cortex are less discriminatory, exciting claustrum neurons projecting to both default mode network nodes and the executive control network nodes [23]. Cingulate inputs to the claustrum may thus guide default mode and executive control network switching [122], which is supported by evidence that anterior cingulate terminals in the claustrum are active at the onset of a cognitively demanding task and required for optimal cognitive performance [44]. In humans, the claustrum is functionally connected to many cortical networks [31,33] and activated immediately prior to deactivation of the default mode network and activation of the executive control network [31]. In humans and rodents, claustrum activity scales with cognitive demand and is required for optimal cognitive control [16,18,45,126]. Altogether, this supports a model wherein the claustrum is activated by executive frontal cortices to instantiate cortical networks supporting cognitive control [41].

The observation of the PSZ1, which predominantly receives inputs from the prefrontal and anterior cingulate cortices and projects to cortical regions in the executive control, default mode, and salience networks, provides the anatomical foundation for cortical networking subserving cognitive control in primates. However, the tracing studies further suggest that the claustrum may more broadly instantiate cortical networks through PSZ2–4 to execute a variety of perceptual and behavioral functions: PSZ2 is positioned to instantiate visual networks; PSZ3 is primed to instantiate the anterior-temporal network involved in memory retrieval; and PSZ4 is anatomically positioned to instantiate the sensorimotor network. The expanded function of claustrum instantiating a variety of cortical networks is supported in rodents by a demonstrated sensorimotor circuit through the claustrum that is functionally separate from prefrontal cortical circuits [30]. In primates, this is supported by data showing that the claustrum is functionally connected to a large number of cortical networks [33,36] and active during a variety of cognitive tasks [47,48].

If the claustrum is comprised of PSZs that each support distinct brain networks, how may the claustrum coordinate activity between networks? Considering that the PSZs occupy largely mutually exclusive territories in the claustrum with minimal communication between PSZs and largely reciprocal anatomical connectivity with the associated cortical areas, it may be reasonable to conclude that the claustrum PSZs function entirely independently. However, PSZ1 exhibits a privileged anatomic architecture, in terms of its cortical connectivity and expanse across the claustrum, that may allow it to guide the activity of other PSZs and associated cortical networks. As mentioned previously, the PSZ1 is positioned to instantiate cortical networks involved in extrinsic and intrinsic cognitive tasks. As such, the PSZ1 may be able to toggle between these extrinsic and intrinsic cognitive states, turning off one cortical network state while turning on the other. Additionally, the territory of PSZ1 overlaps with the territories of PSZ2–4. This may allow PSZ1 to interact with these neighboring PSZs and thus guide the instantiation of more perceptually and behaviorally specific cortical networks as needed.

**Figure 3.**
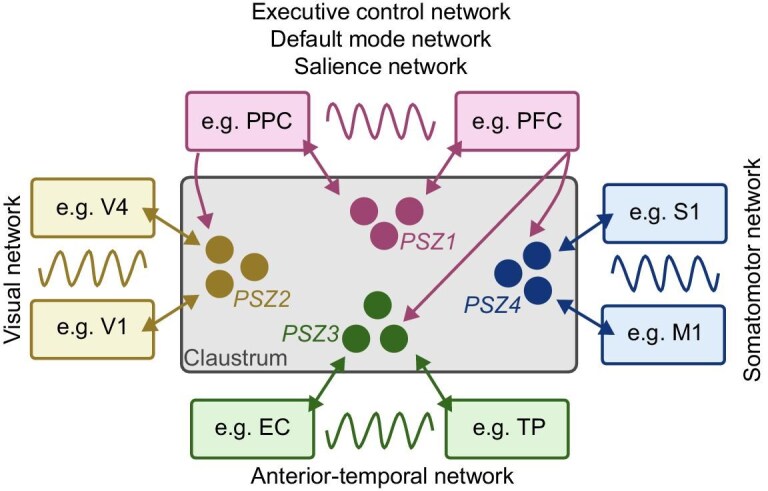
A cortico-claustro-cortical circuit model for PSZ1 control of PSZ1–4 output. Cortical inputs to PSZ1 from prefrontal (PFC) and posterior parietal (PPC) cortices may drive activation of common network-participating neurons within PSZ1. Enabled by the overlap of PSZ1 with PSZ2–4, the same cortical inputs to PSZ1 may activate claustrum projection neurons and/or inhibitory interneurons in the overlapping PSZ2–4, thereby regulating their respective network states. Thus, cognitive network nodes in the cortex (e.g. PPC and PFC) may exert executive influence over other brain networks mediated by PSZ2–4 through their connections with the visual cortex, temporal cortex, and somatosensory and motor cortex, respectively. EC, entorhinal cortex; M1, primary motor cortex; PFC, prefrontal cortex; PPC, posterior parietal cortex; S1, primary somatosensory cortex; TP, temporal pole; V1, primary visual cortex; V4, visual area V4.

We propose that PSZ1 may exert control over PSZ2–4 through two different circuit mechanisms. First, prefrontal and parietal cortical inputs to PSZ1, which overlap PSZ3–4 and PSZ2, respectively, may synapse onto projection neurons or interneurons residing in PSZ2–4 (Fig. 3). This would represent a cortico-claustro-cortical circuit configuration allowing for executive control network regions to dictate PSZ2–4 output and, thereby, influence their respective network states and behavioral output. Such a model would suggest that cortical inputs wire to discrete populations of claustrum projection neurons (and associated inhibitory interneurons) in a manner depending on the claustrum projection neuron cortical target, an architecture that is evinced in the mouse [23,27,30].

**Figure 4.**
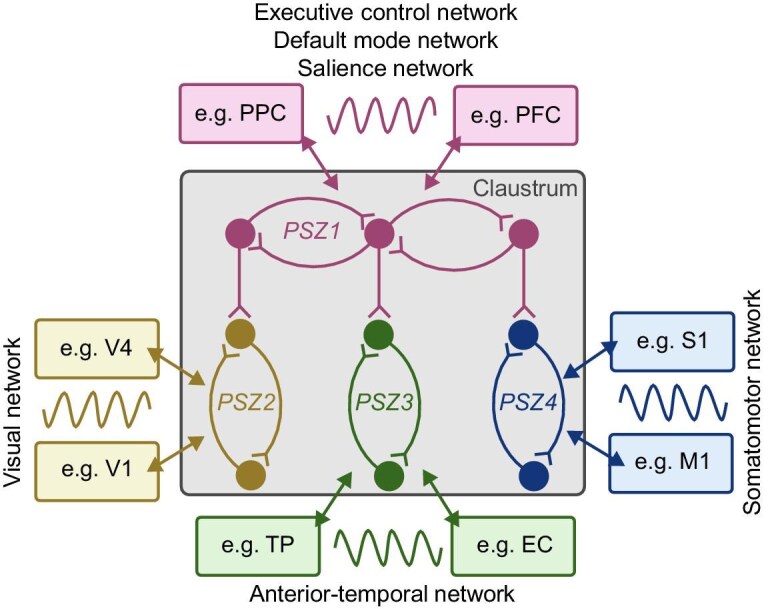
An intra-claustral communication circuit model for PSZ1 control of PSZ1–4 output. In this model, cortical inputs to each PSZ may either activate common network-participating claustrum projection neurons or inhibit (depending on inhibitory microcircuitry) competing network-participating PSZ claustrum projection neurons. Additionally, executive PFC and PPC inputs to PSZ1 may communicate, via excitatory or inhibitory intra-claustral connections, to neurons participating in networks mediated by the overlapping PSZs 2, 3, and 4 to either promote or inhibit (depending on inhibitory microcircuitry) other brain networks through their connections with the visual cortex, temporal cortex, and somatosensory and motor cortex, respectively. EC, entorhinal cortex; M1, primary motor cortex; PFC, prefrontal cortex; PPC, posterior parietal cortex; S1, primary somatosensory cortex; TP, temporal pole; V1, primary visual cortex; V4, visual area V4.

In addition to this cortico-claustro-cortical circuit configuration, the second possible circuit mechanism for PSZ interactions may occur through intra-claustral projections. Previous work in bat and rodent demonstrates that both monosynaptic and polysynaptic excitatory and inhibitory intra-claustral communication exist [8,21,106,107,127]. Corroborating and extending this work in the macaque, Lei and colleagues [74] showed that intra-claustral connections are restricted to each PSZ. Therefore, in this model, claustrum projection neurons activated by the cortex in PSZ1 could dynamically regulate the output of neighboring PSZ1 projection neurons participating in common or competing networks. Similar within-PSZ regulation could be found in PSZ2–4. Again, since PSZ1 overlaps with PSZ2–4, intra-claustral communication stemming from PSZ1 is positioned to also interact with that of PSZ2–4. Claustrum projection neurons activated by the cortex in PSZ1 could dynamically regulate the output of neighboring neurons in overlapping PSZ2–4 that may be participating in their respective networks (Fig. 4).

## CONCLUSION

The study by Lei and colleagues [74] makes tremendous strides in bridging the gap between rodent and human data to clearly define the anatomical boundaries of the macaque claustrum and, in so doing, providing key insight to the anatomical boundaries of the human claustrum, RBC, and endopiriform nucleus. The expanded functional domain organization of the macaque claustrum, reflecting the proto-domain organization in rodents, suggests that the primate claustrum plays a greater role in cognitive control than previously thought by possibly regulating the activity of a wide range of brain networks. This hints at a potential hierarchical model of claustrum PSZ activity wherein PSZ1 instantiates cognitive control networks while guiding further recruitment of task-specific networks through PSZ2–4. This conceptual framework provides several testable hypotheses. As such, the work by Lei and co-authors [74], taken together with landmark rodent and human studies, provides a clear path toward a deeper understanding of how the human brain undergoes brain-wide, dynamic state changes to meet complex internal and external cognitive and behavioral demands in health.

Claustrum involvement in a wide range of brain networks carries important clinical implications. Depression, addiction, schizophrenia, autism, chronic pain, and other neuropsychiatric disorders are characterized by brain network dysfunction [128–131], and mounting evidence links claustrum dysfunction to several of these conditions [46,132,133]. The notion that the claustrum is a novel treatment target is evidenced thus far by behavior-correcting chemogenetic claustrum circuit manipulations in rodents [132,134–139]. More immediately clinically relevant, the claustrum is targeted by psychedelics [9,33,140–143], which have gained traction as potential therapeutics for depression, post-traumatic stress disorder, addiction, and beyond [144–146]. An understanding of how neuromodulation, including by psychedelic drugs, influences claustrum functional domain interactions may thus provide insight for next-generation therapeutic development.
